# The interpositional bypass with a parietal branch of superficial temporal artery graft for symptomatic atherosclerotic anterior cerebral artery stenosis or occlusion

**DOI:** 10.3389/fneur.2024.1361151

**Published:** 2024-04-05

**Authors:** Chuyang Tai, Cong Ling, Tengchao Huang, Baoyu Zhang, Yang Yang, Lei Wei, Haiwan Wu, Ni Mo, Hui Wang, Chuan Chen

**Affiliations:** ^1^Department of Neurosurgery, Third Affiliated Hospital of Sun Yat-sen University, Guangzhou, Guangdong, China; ^2^Department of Radiology, Third Affiliated Hospital of Sun Yat-sen University, Guangzhou, Guangdong, China; ^3^Department of Neurology, Third Affiliated Hospital of Sun Yat-sen University, Guangzhou, Guangdong, China; ^4^Department of Neurosurgery, Yuedong Hospital, Third Affiliated Hospital of Sun Yat-sen University, Meizhou, Guangdong, China

**Keywords:** atherosclerosis, anterior cerebral artery, cerebral blood perfusion, interpositional bypass, stroke, superficial temporal artery graft

## Abstract

**Background:**

For nonmoyamoya patients with anterior cerebral artery (ACA) stenosis or occlusion, whether direct revascularization of the ACA territory can prevent stroke is still unclear. The objective of this study was to investigate the efficacy and safety of a parietal branch of superficial temporal artery-interposed superficial temporal artery-to-ACA bypass (PISAB) for preventing stroke in patients with symptomatic atherosclerotic ACA stenosis or occlusion (SAASO).

**Methods:**

We retrospectively analyzed the data from patients with SAASO who had undergone PISAB in our center between April 2016 and November 2021. The rates of patency, satisfaction (revascularization grades A and B) of bypass, perioperative complications, recurrence of ischemic stroke, changes in bypass flow, and improvements in cerebral blood perfusion were analyzed.

**Results:**

A total of 19 SAASO patients were involved in this study. Sixteen out of 19 (84.2%) patients were free from any cerebral ischemic events after surgery. Only 3 patients (15.8%) had recurrent stroke postoperatively. Two (10.5%) surgery-related complications occurred, including hyperperfusion syndrome and minor stroke. No skin ischemic complications occurred. The average follow-up period was 50.6 ± 18.3 months. The flow rate of the bypass was significantly increased half a year after surgery (56.2 ± 8.0 mL/min vs. 44.3 ± 5.3 mL/min, *p* < 0.001). The ratio of ipsilateral/contralateral mean transit time in the superior frontal gyrus was decreased significantly after bypass (1.08 ± 0.07 vs. 1.23 ± 0.05, *p* < 0.001) and continued to decrease 6 months after surgery (1.05 ± 0.04 vs. 1.08 ± 0.07, *p* = 0.002). The patency rate of PISAB was 94.7% (18/19) 2 years after surgery. The satisfaction rate of bypass was 89.5% (17/19).

**Conclusion:**

The results of this study indicate that PISAB, as a safe superficial bypass, can effectively reduce the risk of stroke in SAASO patients. More precise conclusions will require randomized control studies.

## 1 Introduction

For moyamoya patients, ischemic symptoms due to chronic hypoperfusion of the frontal lobe can be improved by applying various revascularization strategies to the anterior cerebral artery (ACA) territory (1–3). However, for nonmoyamoya patients with ACA stenosis or occlusion (e.g., atherosclerosis), whether augmented flow achieved by direct bypass to the ACA can prevent cerebral ischemic events is still unclear. Direct revascularization for atherosclerotic ACA ischemia has been rarely reported because, before now, cases of symptomatic atherosclerotic ACA stenosis or occlusion (SAASO) that are intractable with medical therapy are uncommon (4). The other reason is that direct revascularization of the ACA branches is technically challenging (5, 6).

Since there are few suitable scalp arteries long enough to reach the ACA, augmenting blood flow to the ACA territory can mostly be achieved by either A3-A3 anastomosis or interpositional bypass (7, 8). A3-A3 anastomosis, which is performed deep in the interhemispheric fissure, is technically demanding and is relatively risky for SAASO patients with only mild symptoms (9). Moreover, in some cases, the A3 segments on each side are anatomically far apart or angled, making A3-A3 anastomosis inapplicable (10, 11). As a kind of superficial bypass, interpositional bypass to the ACA cortical branch is relatively safe, although rarely reported (6, 7). However, more attention must be paid to both maintaining the patency of the lengthened donor artery and avoiding scalp necrosis due to graft harvesting and skin incision (12, 13)

In recent years, a modified interpositional bypass strategy has been used in our center to treat medically intractable atherosclerotic ACA ischemia. A parietal branch of the superficial temporal artery (pSTA) was harvested as the interposed graft to connect the proximal supplying superficial temporal artery (STA) branch, either the frontal branch of the superficial temporal artery (fSTA) or pSTA, and the ACA cortical branches, either the anteromedial frontal branch (AFB), intermediomedial frontal branch (IFB) or posteromedial frontal branch (PFB). This bypass strategy is feasible in almost all cases despite the different developmental conditions of the bilateral STA branches and is unlikely to cause scalp ischemia. Here, we retrospectively study the efficacy and safety of this pSTA-interposed STA-ACA bypass (PISAB) in preventing cerebral ischemic events in SAASO patients. To our knowledge, no similar reports have previously been published.

## 2 Methods

### 2.1 Patient selection

A retrospective analysis of consecutive SAASO patients treated with PISAB between April 2016 and November 2021 at our center was conducted. Exclusion criteria included: (1) patients with moyamoya diseases; (2) young patients without risk factors for atherosclerosis; (3) patients with combined aneurysms; (4) patients with coincident severe stenosis or occlusion of other arteries; (5) patients who had previously undergone cerebral revascularization; and (6) patients with a follow-up (FU) period <2 years or who were lost to FU. This study was approved by the Ethics Committee of the Third Affiliated Hospital of Sun Yat-Sen University (No. SL-II2023-276-01) and was performed in accordance with the ethical standards established by the 1964 Declaration of Helsinki and its later amendments. Written informed consent was obtained from all patients included in the study.

### 2.2 Surgical indications

The surgical indications were as follows: (1) age < 75 years; (2) symptoms of ischemia in the ACA territory occurring in the last 6 months and believed to be caused by occlusion or tandem stenosis of the proximal segments of the ACA (A1 and A2) while receiving dual antiplatelet and statin therapy; (3) symptoms including headache, transient ischemic attack (TIA), aphasia, minor ischemic stroke (including limb numbness and hemiplegia) (14); (4) a modified Rankin scale (mRS) score ≤ 2 and a National Institute of Health stroke scale (NIHSS) score ≤ 3 on admission (15); and (5) computed tomography perfusion (CTP) image showing the ratio of ipsilateral/contralateral mean transit time (I/C MTT) in the superior frontal gyrus >1.13 (16).

### 2.3 Surgical procedures

**Figure 1:**
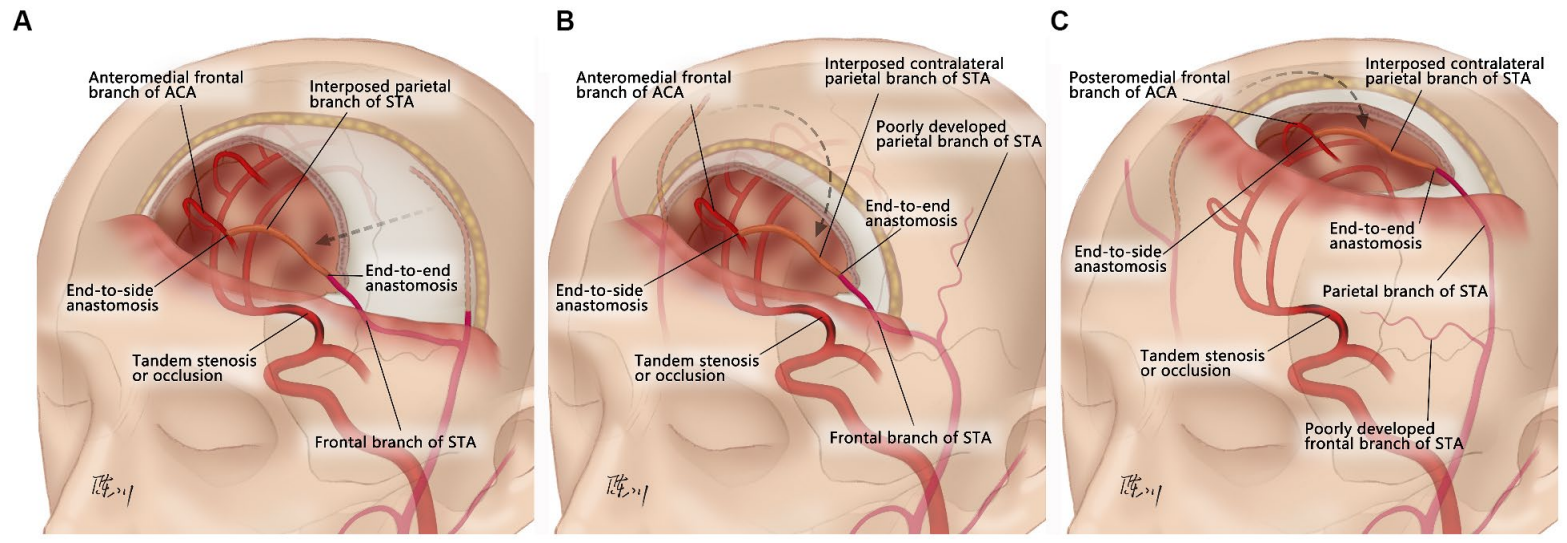
Schematic of the surgical design of the parietal branch of superficial temporal artery-interposed superficial temporal artery-to-anterior cerebral artery bypass. **(A)** fSTA-pSTA-ACA bypass. **(B)** fSTA-cpSTA-ACA bypass. **(C)** pSTA-cpSTA-ACA bypass. fSTA, frontal branch of superficial temporal artery; pSTA, parietal branch of superficial temporal artery; cpSTA, contralateral parietal branch of superficial temporal artery; ACA, anterior cerebral artery.

**Figure 2:**
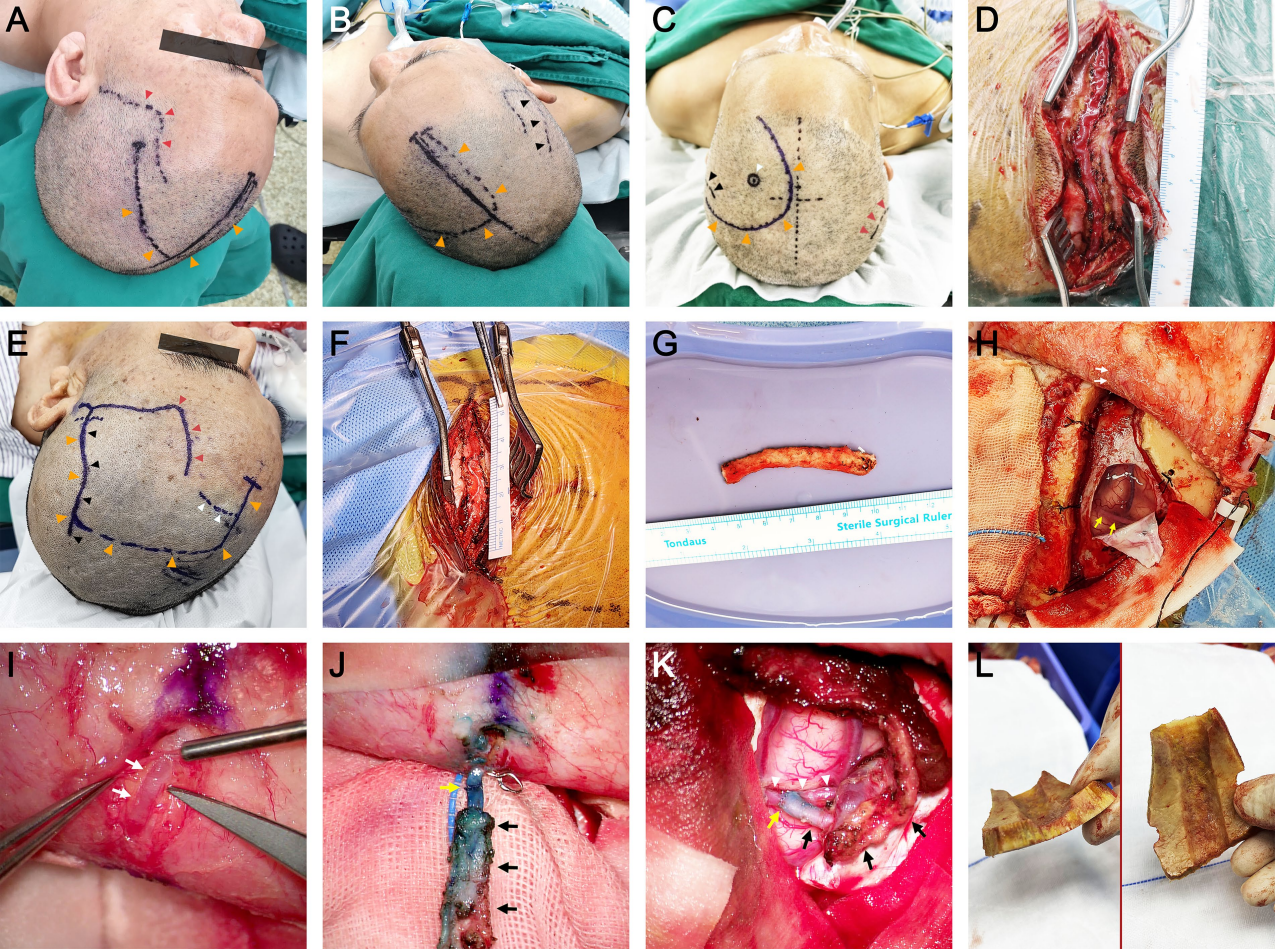
Surgical procedures of the parietal branch of superficial temporal artery-interposed superficial temporal artery-to-anterior cerebral artery bypass. **(A,B)** The design of the skin incision when performing fSTA-cpSTA-ACA bypass. The black arrowheads indicate the course of cpSTA. The orange arrowheads indicate the incision. The red arrowheads indicate the course of fSTA. **(C)** The design of the skin incision when performing pSTA-cpSTA-ACA bypass. The black arrowheads indicate the course of pSTA. The orange arrowheads indicate the incision. The red arrowheads indicate the course of cpSTA. The white arrowhead indicates the site of AFB. **(D)** Dissection of the cpSTA. **(E)** The design of the skin incision when performing fSTA-pSTA-ACA bypass. The black arrowheads indicate the course of pSTA. The orange arrowheads indicate the incision. The red arrowheads indicate the course of fSTA. The white arrowheads indicate the site of AFB. **(F)** Dissection of the pSTA. **(G)** Harvested pSTA graft. **(H)** Craniotomy and dura opening. The white arrows indicate the fSTA (donor artery). The yellow arrows indicate the recipient artery (AFB). **(I)** Dissection of the fSTA from the galeal side. The white arrows indicate a short segment of fSTA. **(J)** End-to-end anastomosis between the fSTA and pSTA grafts. The white asterisk indicates the fSTA stump. The black arrows indicate the pSTA graft. The yellow arrow indicates the anastomosis. **(K)** End-to-side anastomosis between the pSTA graft and AFB. **(L)** The gutter of the bone flap. The white arrowheads indicate the AFB. The black arrows indicate the pSTA graft. The yellow arrow indicates the anastomosis. fSTA, frontal branch of superficial temporal artery; pSTA, parietal branch of superficial temporal; cpSTA, contralateral parietal branch of superficial temporal artery; ACA, anterior cerebral artery; AFB, anteromedial frontal branch. The informed consent for picture publication has been obtained from the patients.

Aspirin (100 mg/d) and atorvastatin (20 mg/d) were prescribed at least 5 days prior to surgery. The patient was placed in the supine position, and general anesthesia was administered. Due to mismatch in the diameter of the ACA cortical branches, neither the radial artery nor the saphenous vein was suitable to be the bypass graft (7, 17). Therefore, pSTA was chosen. When the two branches of the STA on the ipsilateral side were well developed, the pSTA on the ipsilateral side was harvested, and fSTA-pSTA-ACA (AFB or IFB) bypass was performed (Figure 1A). If the pSTA on the ipsilateral side was poorly developed, the contralateral pSTA (cpSTA) was harvested, and fSTA-cpSTA-ACA (AFB or IFB) bypass was performed (Figure 1B). If the fSTA on the ipsilateral side was poorly developed, the cpSTA was harvested, and pSTA-cpSTA-ACA (IFB or PFB) bypass was performed (Figure 1C). Briefly, a straight segment of the pSTA (or cpSTA) was harvested under a microscope, with a length of approximately 5–7 cm. After incising the skin, a craniotomy was performed, and the dura mater was opened to approximately 2 cm x 2 cm. Then, a short segment (approximately 1.0 cm) of the fSTA was dissected from the galeal side. The fSTA and the pSTA (proximal end) were anastomosed end-to-end. Then, the pSTA (distal end) and the AFB (or IFB) were anastomosed end-to-side. When closing the incision, making a gutter in the inner surface of the bone flap is recommended to avoid graft compression (Figure 2). Patients continued to take aspirin (100 mg/day) for their lifetime after surgery.

### 2.4 ACA revascularization assessment

**Figure 3:**
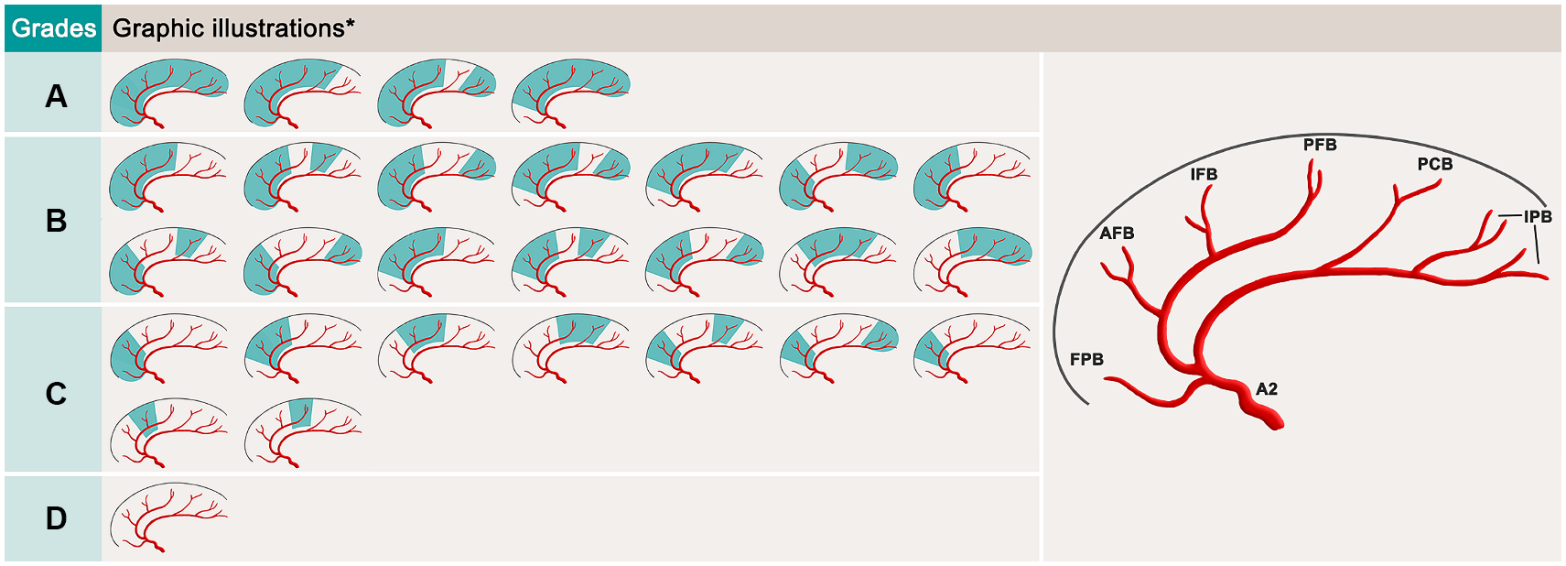
Schematic of the revascularization grading system for the parietal branch of superficial temporal artery-interposed superficial temporal artery-to-anterior cerebral artery bypass. *, the variation in the callosomarginal artery exists. FPB, frontopolar branch; AFB, anteromedial frontal branch; IFB, intermediomedial frontal branch; PFB, posteromedial frontal branch; PCB, paracentral branches; IPB, internal parietal branches.

In this study, a new revascularization grading system, which is based on the amount of ACA cortical branch supplying units perfused by the bypass flow, was proposed to assess the effect of ACA revascularization (Figure 3). A total of 6 ACA cortical branch supplying units were counted in this grading system, including the frontopolar branch unit, AFB unit, IFB unit, PFB unit, paracentral branch unit and internal parietal branch (IPB, including both superior and inferior branches) unit. We defined grade A as when 5 or 6 units were perfused by the bypass flow on postoperative digital subtract angiography (DSA); grade B when 3 or 4 units were perfused; grade C when 1 unit or 2 units were perfused; and grade D when a nonpatent bypass was observed. Both grades A and B indicate satisfactory bypass effects.

### 2.5 Follow-up protocol

The flow rate of the superficial temporal artery trunk (STAt) at the level of the zygoma was detected by Doppler ultrasonography both at the end of the surgery (day 1) and 6 months (day 180) after surgery. Computed tomography angiography (CTA) and CTP were performed on postoperative days 2 and 180 (outpatient FU). DSA was also performed 2 years after surgery to confirm graft patency and revascularization grade. The mRS score and NIHSS score were used to assess the patient’s neurological state.

### 2.6 Statistical analysis

The paired t test was used to compare the flow rate of the STAt at the different FU points and to assess the changes in the I/C MTT ratio. Fisher’s exact tests were used to analyze the enumeration data. *p* < 0.05 was considered to indicate statistical significance. The data were analyzed using SPSS, version 22.0.

## 3 Results

**Figure 4:**
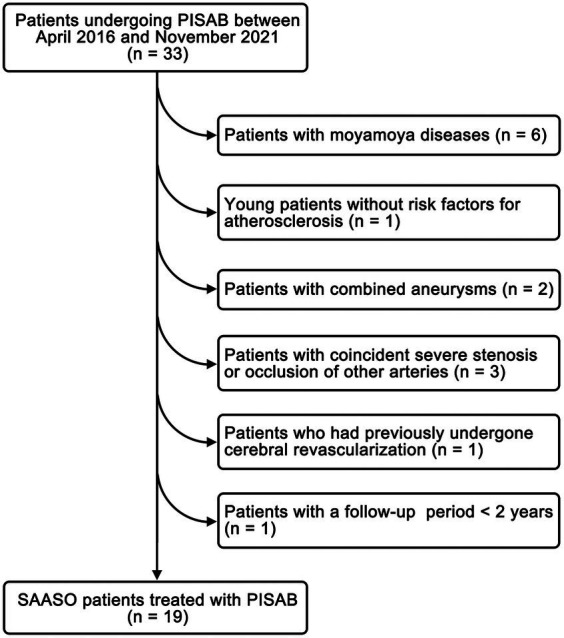
The study flow chart for patient inclusion. PISAB, parietal branch of superficial temporal artery-interposed superficial temporal artery-to-anterior cerebral artery bypass; SAASO, symptomatic atherosclerotic anterior cerebral artery stenosis or occlusion.

A total of 19 patients with SAASO who were treated with PISAB were selected for this study (Figure 4). The patient characteristics and surgery-related information are listed in Table 1. Ten men and 9 women, with an average age of 62.1 ± 7.4 years, were included. The onset symptoms included headache, TIAs, hemiplegia, limb numbness and aphasia. The number of episodes of these symptoms ranged from 1–3 within 6 months before admission. The average I/C MTT ratio in the superior frontal gyrus was 1.23 ± 0.05 (ranging from 1.16 to 1.31) in 19 patients before surgery. A total of 15 patients underwent fSTA-pSTA-ACA bypass, and 3 patients underwent fSTA-cpSTA-ACA bypass. The pSTA-cpSTA-ACA bypass was performed on only 1 patient. The average length of the harvested pSTA graft was 5.7 ± 0.7 cm (ranging from 4.5 to 7.3 cm). The average duration of surgery was 297.1 ± 27.9 min. Two (2/19, 10.5%) complications occurred one week after surgery. One was the onset of seizure combined with headache (case 3), which was considered hyperperfusion syndrome. The other patient (case 14) had a minor stroke in the precentral gyrus that resulted in slight upper limb weakness that recovered in 4 days. No ischemic complications of the skin occurred.

**Table 1:** Patient characteristics and surgery-related information.

Pt. No.	Age (years); Gender	HTN	T2DM	HLP	Smoking	Symptoms	Episodes ≤6 months	ACA lesion	Side of ACA lesion	mRS score	NIHSS score	I/C MTT Ratio	Bypass strategy	Length of graft (cm)	Duration of surgery (min)	Complications
1	57/F	Yes	No	Yes	No	TIA	2	Tandem stenosis	Left	1	0	1.21	fSTA-pSTA-ACA	5.4	319	None
2	64/M	Yes	Yes	No	No	TIA	1	Occlusion	Right	1	0	1.25	fSTA-pSTA-ACA	6.1	289	None
3	55/M	Yes	Yes	Yes	Yes	Hemiplegia	1	Occlusion	Right	2	2	1.3	fSTA-pSTA-ACA	4.5	297	Seizure,headache
4	52/F	No	Yes	Yes	No	TIA	2	Tandem stenosis	Right	0	0	1.19	fSTA-pSTA-ACA	6.3	241	None
5	71/F	Yes	No	Yes	No	TIA, Headache	3	Tandem stenosis	Left	1	0	1.18	fSTA-pSTA-ACA	5.8	289	None
6	68/M	No	Yes	Yes	No	TIA	1	Tandem stenosis	Right	0	0	1.21	fSTA-cpSTA-ACA	5.3	312	None
7	61/M	No	Yes	Yes	Yes	TIA, Headache	2	Occlusion	Right	1	0	1.27	fSTA-pSTA-ACA	4.8	269	None
8	49/M	Yes	No	Yes	Yes	TIA	1	Tandem stenosis	Left	0	0	1.16	fSTA-pSTA-ACA	5.0	299	None
9	74/M	Yes	Yes	Yes	Yes	Aphasia	1	Tandem stenosis	Left	2	1	1.21	fSTA-pSTA-ACA	6.4	335	none
10	61/M	Yes	No	No	Yes	TIA	2	Occlusion	Left	0	0	1.30	fSTA-cpSTA-ACA	6.1	326	None
11	69/F	No	Yes	No	No	TIA	1	Tandem stenosis	Right	0	0	1.25	fSTA-pSTA-ACA	5.8	286	None
12	65/F	Yes	No	No	No	Hemiplegia	3	Tandem stenosis	Left	2	2	1.30	pSTA-cpSTA-ACA	7.3	341	None
13	55/F	Yes	No	Yes	No	TIA	1	Tandem stenosis	Left	0	0	1.24	fSTA-pSTA-ACA	5.4	291	None
14	52/M	Yes	Yes	Yes	Yes	TIA, headache	2	Tandem stenosis	Left	1	0	1.17	fSTA-pSTA-ACA	5.6	263	Minor stroke
15	72/F	Yes	No	Yes	No	Limb numb	1	Tandem stenosis	Right	1	1	1.22	fSTA-pSTA-ACA	4.6	307	None
16	64/F	Yes	Yes	No	No	Limb numb	1	Tandem stenosis	Left	1	1	1.19	fSTA-pSTA-ACA	5.7	274	None
17	59/M	No	Yes	No	Yes	TIA	1	Tandem stenosis	Right	0	0	1.22	fSTA-cpSTA-ACA	6.4	347	None
18	61/M	Yes	No	No	Yes	TIA, headache	2	Tandem stenosis	Left	1	0	1.23	fSTA-pSTA-ACA	6.3	275	None
19	70/F	Yes	Yes	Yes	No	Hemiplegia, aphasia	1	Occlusion	Left	2	3	1.31	fSTA-pSTA-ACA	6.0	285	None

Pt. No., patient number; HTN, hypertension; T2DM, type 2 diabetes mellitus; HLP, hyperlipidemia; ACA, anterior cerebral artery; mRS, modified Rankin scale; NIHSS, National Institute of Health stroke scale; I/C MTT, ipsilateral/contralateral mean transit time; M, male; F, female; TIA, transient ischemia attack; fSTA, frontal branch of superficial temporal artery; pSTA, parietal branch of superficial temporal artery; cpSTA, contralateral parietal branch of superficial temporal artery.

**Figure 5:**
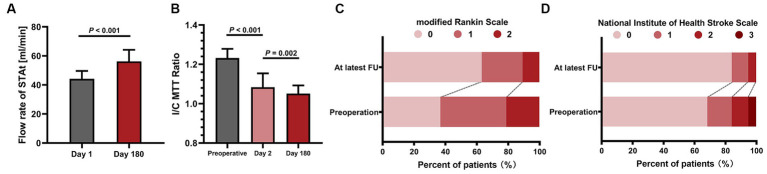
The statistical graphs show the increase in bypass flow and the improvement in both cerebral blood perfusion and neurological state. **(A)** The significant increase in the bypass flow rate 6 months after surgery. **(B)** Continuous improvement in the I/C MTT ratio. **(C)** The improvement of the modified Rankin scale at the latest FU point. **(D)** Improvement in the National Institute of Health Stroke scale score at the latest FU point. STAt, superficial temporal artery trunk; I/C MTT, ipsilateral/contralateral mean transit time; FU, follow-up.

The FU information is listed in Table 2. The average FU period was 50.6 ± 18.3 months. A total of 16 patients (84.2%, 16/19) were free of ischemic events after bypass. One patient suffered minor stroke 3 days after surgery (abovementioned case 14). Another 2 patients (cases 10 and 19) underwent TIA in the FU period. Thus, the rate of stroke recurrence was 15.8% (3 of 19). The flow rate of the STAt on day 180 was significantly increased compared to that on day 1 (56.2 ± 8.0 mL/min vs. 44.3 ± 5.3 mL/min, *p* < 0.001). The I/C MTT ratio in the superior frontal gyrus significantly improved after bypass (1.08 ± 0.07 vs. 1.23 ± 0.05, *p* < 0.001) and continued to significantly decrease 6 months after surgery (1.05 ± 0.04 vs. 1.08 ± 0.07, *p* = 0.002). No graft occlusion was observed on CTA scan 6 months after bypass. However, one patient (case 10) with bypass nonpatency was identified on DSA 2 years after surgery. Thus, the patency rate 2 years after bypass was 94.7% (18/19) in this group. According to the DSA results 2 years after surgery, 10 patients had grade A revascularizations, 7 had grade B, 1 had grade C and 1 had grade D, and the satisfaction rate of the bypass effect was 89.5% (17/19). During the FU period, both the overall mRS score and the overall NIHSS score were improved at the last FU (Figure 5).

**Table 2:** The follow-up information.

Pt. No.	FU period (month)	Ischemic events ≤30 Days	Ischemic events >30 Days	Flow rate of STAt on Day 1 (mL/min)	Flow rate of STAt on Day 180 (mL/min)	I/C MTT ratio on Day 2	I/C MTT ratio on Day 180	Bypass patency on Day 180	Bypass patency 2 Years after surgery	Revascularization grade	mRS Score at Latest FU	NIHSS score at latest FU
1	86	None	None	41	51	1.08	1.04	Yes	Yes	B	0	0
2	80	None	None	51	59	1.03	1.05	Yes	Yes	A	0	0
3	71	None	None	57	72	1.1	1.04	Yes	Yes	A	2	1
4	70	None	None	39	60	0.98	1.01	Yes	Yes	A	0	0
5	65	None	None	44	61	1.08	1.02	Yes	Yes	B	0	0
6	62	None	None	46	59	1.03	0.98	Yes	Yes	A	0	0
7	59	None	None	44	49	1.13	1.10	Yes	Yes	A	1	0
8	55	None	None	53	61	1.01	1.03	Yes	Yes	B	0	0
9	53	None	None	43	48	0.99	1.02	Yes	Yes	A	1	0
10	49	None	TIA	40	43	1.20	1.12	Yes	No	D	1	0
11	44	None	None	43	51	1.16	1.07	Yes	Yes	A	0	0
12	43	None	None	37	47	1.17	1.11	Yes	Yes	C	1	0
13	41	None	None	38	50	1.09	1.06	Yes	Yes	B	0	0
14	39	Minor stroke	None	43	62	1.05	1.03	Yes	Yes	A	0	0
15	33	None	None	41	59	1.09	1.02	Yes	Yes	A	1	1
16	32	None	None	41	48	0.98	1.01	Yes	Yes	B	0	0
17	28	None	None	48	70	1.12	1.06	Yes	Yes	B	0	0
18	27	None	None	42	55	1.09	1.06	Yes	Yes	A	0	0
19	25	None	TIA	50	63	1.21	1.14	Yes	Yes	B	2	2

Pt. No., patient number; FU, follow-up; STAt, superficial temporal artery trunk; mRS, modified Rankin scale; NIHSS, National Institute of Health stroke scale; I/C MTT, ipsilateral/contralateral mean transit time; TIA, transient ischemia attack.

## 4 Discussion

Due to the rarity of the condition and the technical difficulty of its surgical treatment, cases in which nonmoyamoya patients with isolated SAASO have been treated with superficial bypass surgeries have been reported far less often (18). The few studies that have been published thus far are case reports (4, 19–22). In this study, the clinical data of a series of 19 SAASO patients who were treated with PISAB were retrospectively analyzed, and the results revealed that this modified bypass technique was both efficacious and safe. To our knowledge, the present study is the first case series to estimate the efficacy and safety of PISAB in treating nonmoyamoya ACA stenosis or occlusion.

### 4.1 Efficacy

**Figure 6:**
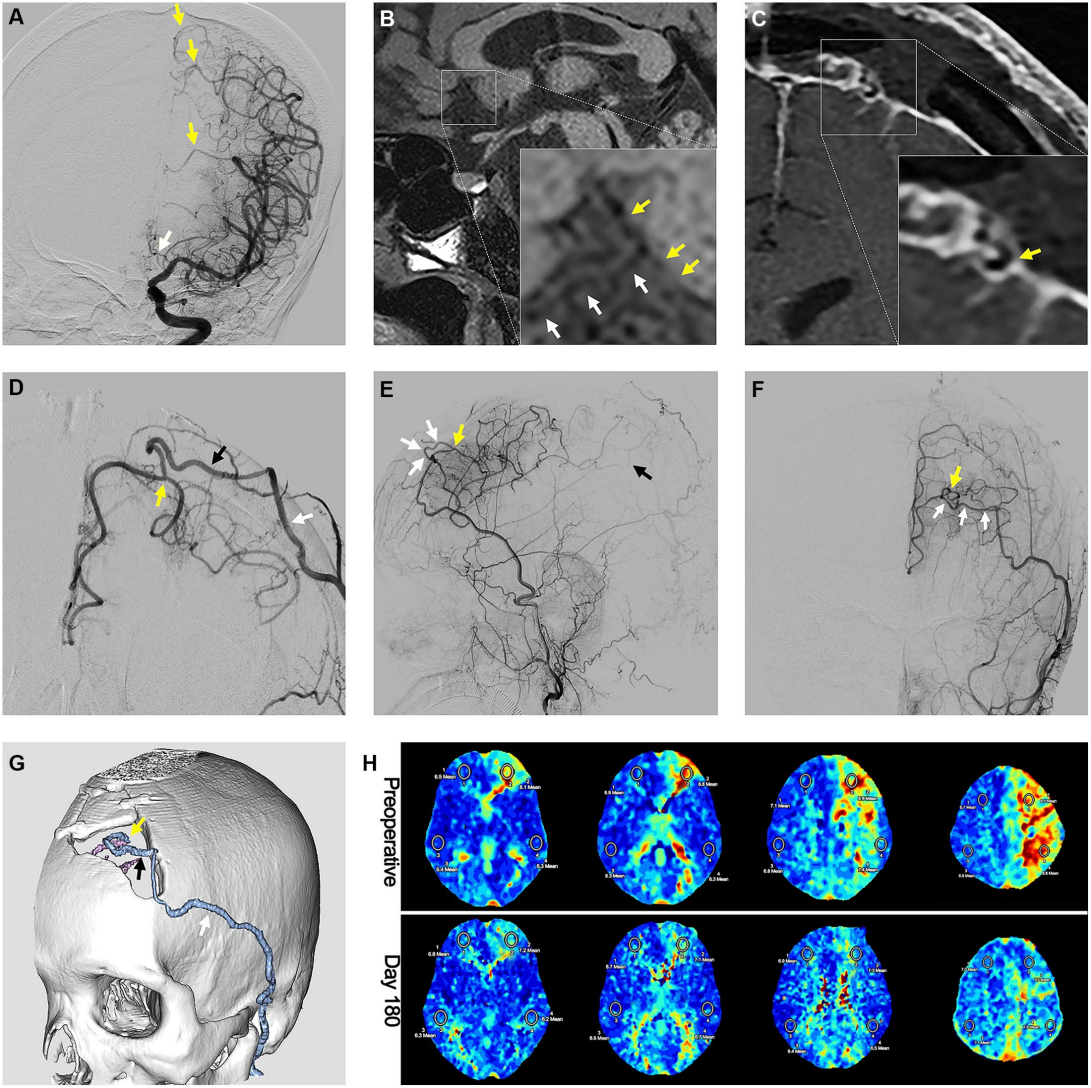
Preoperative and postoperative radiological images showing the efficacy of the parietal branch of superficial temporal artery-interposed superficial temporal artery-to-anterior cerebral artery bypass. **(A)** Preoperative DSA image showing the occlusion of the ACA. The yellow arrows indicate the compensation flow from cortical branches of the middle cerebral artery. The white arrow indicates the occlusion of ACA. **(B)** High-resolution MRI image showing atherosclerotic occlusion of the A2 segment. The yellow arrows indicate the atherosclerotic occlusion. The white arrows indicate the orbitofrontal branch. Postoperative **(C)** high-resolution MRI image and **(D)** DSA image showing the anastomosis of the pSTA and ACA. The yellow arrow indicates the anastomosis. The black arrow indicates the pSTA graft. The white arrow indicates the fSTA. Postoperative DSA images in **(E)** A-P view and **(F)** lateral view showing the ACA blood flow brought by bypass graft. The yellow arrow indicates the anastomosis. The white arrows indicate the pSTA graft. The black arrow indicates the inferior branch of internal parietal branches. **(G)** Computed tomography angiography showing the course of the pSTA graft. The yellow arrow indicates the anastomosis. The black arrow indicates the pSTA graft. The white arrow indicates the fSTA. **(H)** Perfusion improvement in the frontal lobe displayed by the mean transit time sequence of MRI. DSA, digital subtract angiography; ACA, anterior cerebral artery; MRI, magnetic resonance imaging; pSTA, parietal branch of superficial temporal artery. fSTA, frontal branch of superficial temporal artery.

In the FU period (average 50.6 ± 18.3 months), 16 out of 19 (84.2%) SAASO patients were free from any cerebral ischemic events after PISAB. Only 3 patients (15.8%) had ischemic events after revascularization. One patient experienced a minor stroke 3 days after surgery. Magnetic resonance imaging indicated a small new infarction in the ipsilateral precentral gyrus that was distant from the anastomosis site. Thus, the flow conflict between the STA graft flow and the preexisting compensation blood flow was considered the cause, and upper limb weakness completely resolved in 4 days. The other two patients suffered a TIA in the FU period. One TIA was probably due to graft occlusion, and the other may have been caused by an unstable underlying disease. In addition, cerebral blood perfusion (CBP) in the ipsilateral frontal lobe continued to improve, and the patients’ neurological states (mRS score and NIHSS score) improved after surgery. Additionally, the bypass grafts in 94.7% (18/19) of patients remained patent 2 years after surgery, and 89.5% (17/19) of patients had a satisfactory bypass effect. All these results initially suggested the potential effectiveness of PISAB in reducing the risk of stroke in SAASO patients (Figure 6).

### 4.2 Safety

Due to the small diameter and tortuous morphology of proximal ACA segments (A1 and A2), interventional therapy is a high risk for SAASO patients (23). Creating an A3-A3 anastomosis is technically demanding but is also applicable for SAASO patients. However, the deep anastomosis in the interhemispheric fissure makes this prophylactic bypass relatively risky (e.g., postoperative hemorrhage) for patients with only mild ischemic symptoms (9, 10). Comparatively, PISAB is a safer operation. Due to the small dura opening and both the low-flow and superficial nature, PISAB is less likely to cause brain damage or hyperperfusion hemorrhage. Thus, no postoperative hemorrhagic events were observed in this group. However, there were two (10.5%, 2/19) bypass-related complications. One complication (case 14) was a minor stroke caused by flow conflict. The other patient (case 3) suffered headache and seizures after surgery, and the postoperative flow rate of STAt was the highest in this group. Thus, hyperperfusion syndrome was considered the cause, and the symptoms were significantly relieved by lowering the systolic blood pressure and infusing mannitol. None of these complications resulted in any permanent neurological defects. In addition, ischemic complications of the skin were commonly considered and encountered in previous studies due to the dissection or harvest of both STA branches to create interpositional bypass (12, 13). However, there were no wound complications in our study. This is because in the bypass strategies we adopted, the supplying STA branch (mostly the fSTA) was dissected only 1 cm at the distal end, whereas the grafted pSTA branch was obtained either from the ipsilateral side but via an incision exactly along its course or from the contralateral side. Thus, our incision designs have less impact on ipsilateral scalp blood supply.

### 4.3 Surgical techniques

There are several surgical tips that need attention to promote the patency and safety of PISAB. First, among the available recipient arteries (AFB, IFB and PFB), we suggest choosing AFB and IFB if possible. This is because the PFB is mostly near the precentral gyrus, and the nearby drainage veins are relatively thick. Damage to these structures might cause neuro-functional impairment. Since the fSTA is naturally closer to both the AFB and IFB than to the PFB, we tend to choose the fSTA as the feeding artery. Second, the pSTA graft should be fully flushed with heparin saline immediately after being harvested to prevent thrombosis inside (Supplementary Video S1). Third, the proximal stump of the pSTA graft should be trimmed into a fish-mouth shape to prevent anastomosis stenosis or occlusion. Fourth, it is important to avoid compression of the pSTA graft. A gutter made by a drill on the bone flap is helpful to keep the graft patent (24). Finally, compared with the cortical branches of the MCA, the cortical branches of the ACA are more distal and thinner, and thus, the blood flow compensation in the cortical territory of the ACA is poorer (especially in cases of ACA occlusion). Therefore, during anastomosis, the clipping duration of ACA cortical branches should be shortened as much as possible to avoid the occurrence of iatrogenic ischemic events.

### 4.4 Revascularization grading system

Since no assessment system for ACA revascularization is currently available, we proposed a new revascularization grading system to assess the efficacy of PISAB. Among all the cortical branches of the ACA, only the orbitofrontal branch (OFB) was excluded from this system. This is because the OFB is the most proximal branch, and the opening of the OFB is usually involved in stenosis or occlusion of the A2 segment. Thus, this branch, in most cases, cannot be reached by bypass flow. In addition, the superior and inferior branches of IPB are anatomically close, and both originate mostly from the terminus of the pericallosal artery, so we categorize them into the same branch unit. Unlike the cortical branches of the MCA, the cortical branches of the ACA are all located neatly in the interhemispheric space of the brain, so they can be clearly shown in the lateral view of DSA images. Therefore, counting the number of branch supplying units that are irrigated by the bypass flow in the lateral view of DSA images is relatively simple, and this branch-unit-based grading system is also much clearer and more precise than roughly dividing the revascularization efficacy into 1/3 or 2/3 of the entire ACA supplying area.

### 4.5 Limitations

Although this study is limited by its small sample size, retrospective nature and single institution design, it nonetheless represents, to our knowledge, the largest case series dedicated to STA-ACA interpositional bypass for nonmoyamoya ACA stenosis or occlusion. In addition, the bypass strategy described herein is technically challenging and currently performed in only a few large centers, which limits its generalizability. Finally, this study exclusively included Chinese patients.

## 5 Conclusion

This study indicated that PISAB can continuously improve CBP and effectively reduce the risk of stroke in SAASO patients. Moreover, this modified revascularization strategy, as a low-flow and cortical bypass with limited dura opening, can be safely performed. Additionally, with the incision design of PISAB, scalp ischemic complications can be avoided. More precise conclusions will require randomized control studies.

## Data availability statement

The raw data supporting the conclusions of this article will be made available by the authors, without undue reservation.

## Ethics statement

The studies involving humans were approved by the Ethics Committee of the Third Affiliated Hospital of Sun Yat-Sen University. The studies were conducted in accordance with the local legislation and institutional requirements. The participants provided their written informed consent to participate in this study.

## Author contributions

CT: Formal analysis, Methodology, Writing – original draft. CL: Formal analysis, Methodology, Writing – original draft. TH: Formal analysis, Methodology, Writing – original draft. BZ: Data curation, Writing – review & editing. YY: Data curation, Writing – review & editing. LW: Writing – review & editing, Visualization. HaW: Writing – review & editing, Visualization. NM: Writing – review & editing. HuW: Conceptualization, Supervision, Writing – review & editing. CC: Conceptualization, Funding acquisition, Supervision, Writing – review & editing.
